# Effectiveness of active occupational therapy in patients with acute stroke: A propensity score-weighted retrospective study

**DOI:** 10.3389/fresc.2022.1045231

**Published:** 2023-01-05

**Authors:** Shiori Yamakawa, Hirofumi Nagayama, Kounosuke Tomori, Kohei Ikeda, Ayaka Niimi

**Affiliations:** ^1^ Department of Occupational Therapy, Kinugasa Hospital, Yokosuka, Japan; ^2^ Department of Occupational Therapy, Kanagawa University of Human Services, Yokosuka, Japan; ^3^ Department of Occupational Therapy, Tokyo University of Technology, Tokyo, Japan; ^4^ Department of Occupational Therapy, Yokohama Brain and Spine Center, Yokohama, Japan

**Keywords:** occupational therapy (OT), stroke rehabilitaiton, recovery of function/prognosis, acute care, physical therapy (PT)

## Abstract

**Background and purpose:**

The effects of therapy and patient characteristics on rehabilitation outcomes in patients with acute stroke are unclear. We investigated the effects of intensive occupational therapy (OT) on patients with acute stroke.

**Methods:**

We performed a retrospective cohort study using the 2005–2016 Japan Rehabilitation Database, from which we identified patients with stroke (*n* = 10,270) who were admitted to acute care hospitals (*n* = 37). We defined active OT (AOT) and non-AOT as OT intervention times (total intervention time/length of hospital stay) longer or shorter than the daily physical therapy intervention time, respectively. The outcomes assessed were the Functional Independence Measure (FIM) and National Institutes of Health Stroke Scale (NIHSS) scores, duration of hospitalization, and rate of discharge. Propensity scores and inverse probability of treatment weighting analyses adjusted for patient characteristics were performed to investigate the effects of AOT on patient outcomes.

**Results:**

We enrolled 3,501 patients (1,938 and 1,563 patients in the AOT and non-AOT groups, respectively) in the study. After inverse probability of treatment weighting, the AOT group had a shorter length of hospitalization (95% confidence interval: −3.7, −1.3, *p* < 0.001), and the FIM (95% confidence interval: 2.0, 5.7, *p* < 0.001) and NIHSS (95% confidence interval; 0.3, 1.1, *p* < 0.001) scores improved significantly. Subgroup analysis showed that lower NHISS scores for aphasia, gaze, and neglect and lower overall NIHSS and FIM scores on admission led to a greater increase in FIM scores in the AOT group.

**Conclusions:**

AOT improved the limitations in performing activities of daily living (ADL) and physical function in patients with acute stroke and reduced the length of hospitalization. Additionally, subgroup analysis suggested that the increase in FIM score was greater in patients with severe limitations in performing ADLs and worse cognitive impairment, such as neglect, on admission.

## Introduction

Stroke is associated with a significant burden of care worldwide; 43% of elderly patients with stroke have moderate to severe neurological deficits (1, 2). Rehabilitation after stroke is associated with a reduced incidence of disability and mortality, and several guidelines recommend initiation of rehabilitation during the acute phase of stroke (3, 4). Additionally, recent meta-analyses have reported that early rehabilitation after stroke improves the limitations in performing activities of daily living (ADLs) (5).

Researchers have previously investigated the intensity and timing of the initiation of rehabilitation for patients with acute stroke (6–11). Performing at least 3 h of high-intensity rehabilitation between 24 and 48 h after stroke onset can reportedly improve the modified Rankin (mRankin) score 3 months after onset (9). In these studies, the interventions (1) commenced within 24 h of stroke onset, (2) focused on out-of-bed activities, such as sitting, and (3) provided at least three out-of-bed activities in addition to usual care (6–9). These interventions were provided by a physical therapist or nurse, but some studies did not differentiate between physical and occupational therapists. Furthermore, the interventions did not include direct ADLs (e.g., dressing exercises) performed by occupational therapists. Therefore, fundamental questions regarding the type of therapy and patient characteristics on rehabilitation outcomes remain unanswered (9).

In clinical practice, physical therapy, occupational therapy (OT), and speech and language therapy are often provided to patients with acute stroke. In a recent systematic review, OT during the acute phase of stroke effectively improved limitations in performing ADLs, reduced symptoms of delirium, and improved cognitive function, although with limitations (12). Further, the role of occupational therapists in the intensive care unit is not well established, behooving occupational therapists to expand their role and lead original research (13). Although most patients received physical therapy, OT was not widely implemented for patients with acute stroke (14). However, we believe that OT may effectively improve the quality of life of patients with acute stroke. Thus, we hypothesized that active and high intensity OT would improve the limitations in performing ADLs in patients with stroke.

The purpose of this study was to investigate the effects of active OT (AOT) on patients with acute stroke. Additionally, we conducted a subgroup analysis to determine which patient characteristics were associated with the efficacy of AOT.

## Materials and methods

We performed a retrospective cohort study using information from the Japan Rehabilitation Database (15, 16). The need for informed consent was waived because all data were de-identified. The study was approved by the ethics committee of the Kanagawa University of Human Services (No. 7-20-30).

### Data source

We retrospectively obtained data from the Japan Rehabilitation Database, which included voluntary sampling data collected from patients admitted to participating hospitals between January 2005 and March 2016 (15–17). The data are divided into various sections depending on the diagnosis and stroke phase such as stroke in the medical ward, stroke in the convalescent rehabilitation ward, and other conditions. The stroke database includes patient characteristics, such as age, stroke type, and severity based on the National Institutes of Health Stroke Scale (NIHSS) and Functional Independence Measure (FIM) scores, type of rehabilitation, and rehabilitation time provided. As of 2016, 80 hospitals were participating and data from 33,657 patients had been accumulated. In this study, we used all data collected between the time of admission and discharge of patients with stroke admitted to acute care hospitals (37 hospitals, *N* = 10,270).

### Patients

Patients who were included in the study were as follows: those with a first episode of stroke, those who independently performed ADLs before stroke onset (mRankin score of 0 or 1), those hospitalized directly due to stroke (onset did not occur during hospitalization), those living at home before stroke onset, and those hospitalized within 7 days following stroke onset. The exclusion criteria were as follows: those who died during hospitalization, those whose duration of hospitalization could not be confirmed, those who did not receive confirmed rehabilitation during hospitalization, those who were hospitalized for over 180 days, and those who received over 180 min of OT, physical therapy, or speech therapy individually.

### Intervention

The patients were divided into the AOT and non-AOT groups. AOT and non-AOT were defined as daily OT intervention times (total intervention time/length of hospital stay) longer or shorter than the daily physical therapy intervention times, respectively. Reportedly, occupational therapy places emphasis on increasing upper-extremity control and improving performance of basic ADLs (18). Additionally, acute phase occupational therapy is provided on an individualized basis and addresses training and re-education in ADLs, assessment of assistive devices, training in use, and support for discharge (12). Supplemental interventions include cognitive stimulation, multi-sensory stimulation, and positioning techniques, along with family and/or primary caregiver education or visits to the patient's home with subsequent environmental assessments (12). Therefore, it was expected that more of these interventions would be provided in the AOT group.

### Outcomes

The primary outcome was an increase in the FIM score (FIM score at discharge minus FIM score at admission) as a measure of improvement in performing ADLs before and after the intervention. The FIM is widely used to assesses limitations in performing ADLs based on the amount of assistance required to perform basic physical and cognitive activity functions. It consists of 18 items that assess motor (13) and cognitive (5) functions. The total score ranges from 18 to 126, with higher scores indicating better functional status (19, 20).

The secondary outcomes were an improvement in the NIHSS scores (NIHSS value at admission minus NIHSS value at discharge), length of hospitalization (days), and rate of discharge to home. The NIHSS is a reliable tool that is widely used to determine stroke severity in emergency departments (21, 22). It consists of 15 items that assess the following: level of consciousness, eye movements, integrity of visual fields, facial movements, upper and lower extremity strength, sensation, coordination, language, speech, and neglect. Each impairment is rated based on an ordinal scale ranging from 0 to 2, 3, or 4. Scores for each item are added to obtain a total score ranging from 0 to 42, with higher scores indicating greater stroke severity.

### Multiple imputation

We used the multiple imputation method to replace variables with missing values (including the outcome variables) (23). We created 20 imputed datasets using multivariate imputation by chained equations and the “mi impute chained” syntax in Stata (24). The variables used to estimate the substitution value were as follows: age, total FIM scores on admission and at discharge, FIM score on admission, mRankin scale scores on admission and at discharge, Glasgow coma scale (GCS) scores on admission and at discharge, NIHSS scores on admission and at discharge; time before initiating rehabilitation after admission (days); and severity of aphasia, gaze, and neglect. Propensity scores and treatment effects were estimated for each of the 20 datasets.

### Inverse probability of treatment weighting (IPTW)

To reduce the chance of confounding due to non-random assignment to the treatment group, propensity scores were used to balance the distribution of patient characteristics of all the treatment groups at baseline (25, 26). We estimated the propensity scores for all participants in the intervention and control groups using logistic regression analysis within each multiple-imputed dataset (27). When estimating the propensity score, we identified potential confounding factors that may influence the main outcome (FIM score), secondary outcome (NIHSS score), length of hospitalization, and discharge rate based on clinical experience (28). The covariates used to estimate the propensity score were as follows: sex, age, time from stroke onset to admission (days), time to initiation of rehabilitation after admission (days), mRankin, Glasgow coma scale, and NIHSS scores, total FIM score on admission, type of stroke, treatment with recombinant tissue plasminogen activator (yes or no), surgery after hemorrhagic stroke (yes or no), surgery after subarachnoid hemorrhage (yes or no), type of anticoagulant therapy, number of caregivers, affected sides, severity of aphasia, gaze, and neglect according to the NIHSS, and speech and language therapy (yes or no). Additionally, the covariates used to estimate the propensity score must consist solely of pre-treatment covariates (29); therefore, those that occurred after interventions, such as daily rehabilitation time, could not be included in the propensity score estimation. However, when the covariates were unbalanced among the groups, they were adjusted for multiple regression analysis.

We calculated the stabilized IPTW of the observed group using the estimated propensity score to reduce variability in each group and reduce the influence of outliers (30). Each patient was weighted as follows: AOT group, proportion of the AOT group*1/propensity score; non-AOT group, proportion of the non-AOT group*1/(1 – propensity score). We assessed the balance of covariates between the AOT and non-AOT groups by calculating the standardized differences, where a value of <0.1 indicated good balance (31). However, when calculating the propensity score using the variables listed above, we found that the absolute value of the standardized differences for the total FIM score on admission was 0.136. Therefore, based on the literature, we chose a standardized difference of 0.15 rather than 0.1 before conducting our final analyses (32–34).

### Statistical analysis

We compared the baseline characteristics of the eligible patients using two-tailed independent *t*-tests for continuous data and *χ*^2^ tests for categorical data before performing multiple imputation. We then summarized the pre- and post-IPTW baseline characteristics of the patients in both groups by calculating the standardized differences.

For the outcome assessment, we first calculated the mean and standard error of each variable before and after IPTW. Next, the outcomes were compared using multiple regression analysis after IPTW adjustment for unbalanced factors (variables with a standardized difference >0.1 after IPTW were excluded from the propensity score calculation). We conducted a subgroup analysis and examined interactions after IPTW to explore the patient characteristics associated with effective AOT. We unified the pooling of treatment effects for all analyses by averaging the dataset values and estimating standard error based on Ruben's rule and using the “mi estimate: bin- reg” syntax in Stata (27, 28, 35). Stata15.1 (Stata Corp, College Station, TX, United States) was used for all analyses, including the calculation of propensity score, and the significance level was set at *p* < 0.05.

## Results

### Baseline patient characteristics

**Figure 1:**
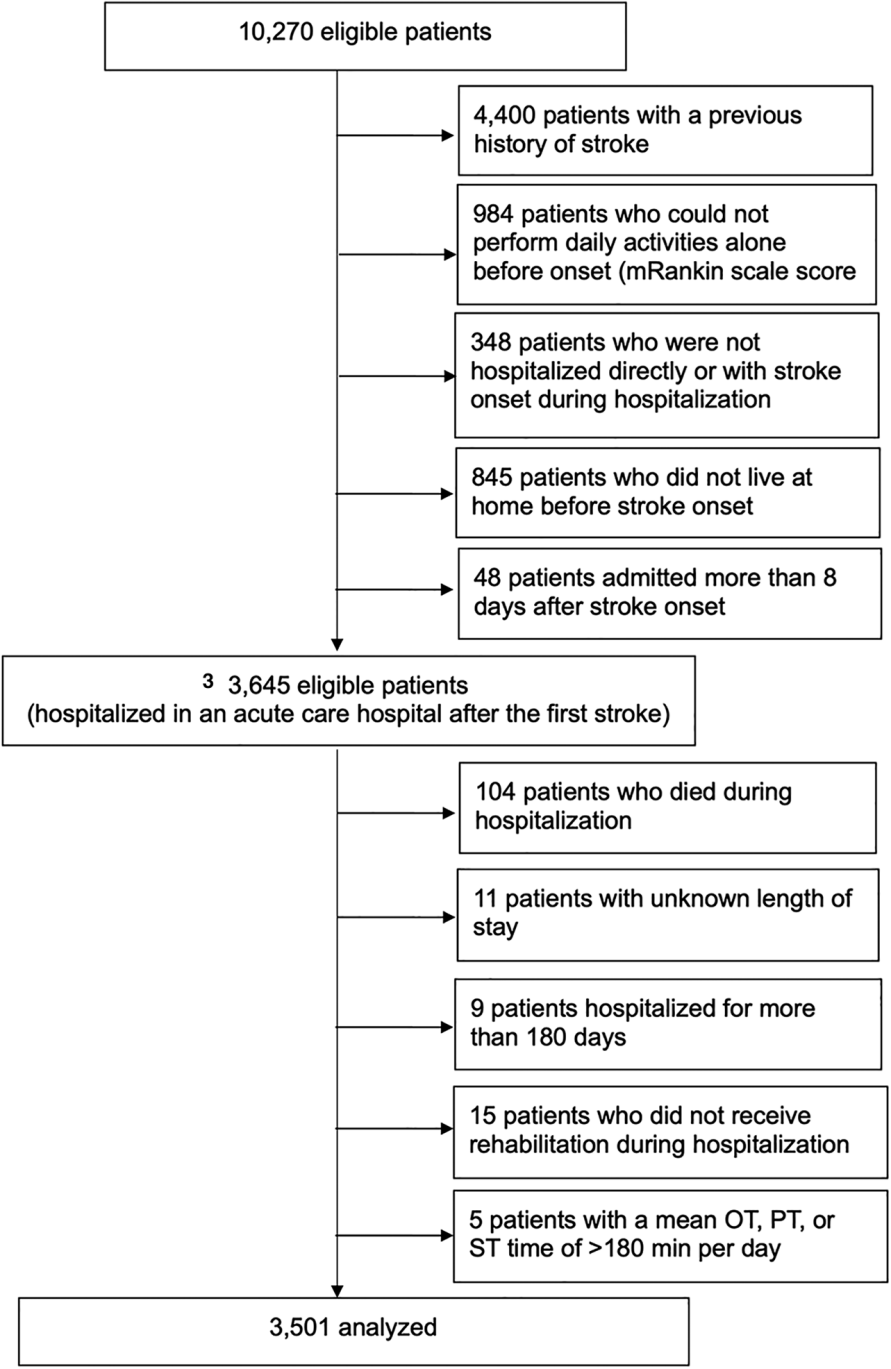
Study flowchart and patient selection. OT, occupational therapy; PT, physical therapy; ST, speech therapy. Final analysis patients were divided into the AOT and non-AOT groups. AOT and non-AOT were defined as daily OT intervention times (total intervention time/length of hospital stay) longer or shorter than the daily physical therapy intervention times, respectively.

After applying the inclusion and exclusion criteria of this study, 3,501 participants were included in the analysis (Figure 1), with 1,563 assigned to the AOT group and 1,938 to the non-AOT group. Table 1 shows the baseline characteristics of the eligible patients before multiple imputation. The variable with the most missing values was the total FIM score on admission (18.0%). There were significant differences between the AOT and non-AOT groups at almost all baseline variables. Table 2 presents the baseline characteristics before and after IPTW after multiple imputations. Before IPTW, the standardized difference of both groups was >0.15 for several variables, indicating significant differences in clinical characteristics, demographics, functional level, and stroke severity. After IPTW, all standardized differences in the weighted comparisons were <0.15, indicating a similar distribution of baseline characteristics between the two groups (Table 2 and Supplementary Figures S1, S2). However, to maintain robustness, a standardized difference of >0.1 for the total FIM score was used as an adjustment factor in the final analysis.

**Table 1:** Characteristics of eligible patients before multiple imputation.

	Total	Non-Active occupational therapy group	Active occupational therapy group	*p*-value
	*N* = 3,501	*N* = 1,563	*N* = 1,938	
Continuous variable, Mean (SD)				
Age	69.2 (13.2)	70.2 (13.2)	68.3 (13.1)	<0.001
Time from onset to admission (days)	1.3 (0.8)	1.3 (0.7)	1.4 (0.9)	0.003
Time to start of rehabilitation after admission (days)	2.7 (6.9)	3.0 (6.9)	2.5 (6.9)	0.040
Missing, *N* (%)	6 (0.2%)	2 (0.1%)	4 (0.2%)	
mRankin scale score	3.6 (1.4)	3.8 (1.4)	3.4 (1.4)	<0.001
Missing, *N* (%)	58 (1.7%)	36 (2.3%)	22 (1.1%)	
^a^GCS score	13.6 (2.7)	13.1 (3.3)	14.0 (2.1)	<0.001
Missing, *N* (%)	25 (0.7%)	15 (1.0%)	10 (0.5%)	
NIHSS score	6.9 (8.2)	8.7 (9.6)	5.4 (6.6)	<0.001
Missing, *N* (%)	35 (1.0%)	22 (1.4%)	13 (0.7%)	
Total FIM score	61.7 (32.7)	54.6 (33.4)	67.6 (30.9)	<0.001
Missing, *N* (%)	629 (18.0%)	263 (16.8%)	366 (18.9%)	
Category variable, *N* (%)				
Female	1,415 (40.4%)	688 (44.0%)	727 (37.5%)	<0.001
Type of stroke				
Ischemic stroke	2,152 (61.5%)	899 (57.5%)	1,253 (64.7%)	<0.001
Hemorrhagic stroke	864 (24.7%)	416 (26.6%)	448 (23.1%)	
Subarachnoid hemorrhage	301 (8.6%)	163 (10.4%)	138 (7.1%)	
Others/Unknown	184 (5.3%)	85 (5.4%)	99 (5.1%)	
^b^rtPA treatment (yes)	77 (2.2%)	49 (3.1%)	28 (1.4%)	<0.001
Surgery after hemorrhagic stroke (yes)	226 (6.5%)	129 (8.3%)	97 (5.0%)	<0.001
Surgery after subarachnoid hemorrhage (yes)	121 (3.5%)	67 (4.3%)	54 (2.8%)	0.016
Anticoagulant therapy				
No	2,523 (72.1%)	1,079 (69.0%)	1,444 (74.5%)	0.002
Anticoagulant therapy (yes)	329 (9.4%)	163 (10.4%)	166 (8.6%)	
Antiplatelet therapy (yes)	330 (9.4%)	151 (9.7%)	179 (9.2%)	
Anticoagulant and Antiplatelet therapy	85 (2.4%)	44 (2.8%)	41 (2.1%)	
Unknown	234 (6.7%)	126 (8.1%)	108 (5.6%)	
Number of Caregivers				
Almost none	1,005 (28.7%)	481 (30.8%)	524 (27.0%)	0.020
Intermediate Almost none and one full-time caregiver	1,423 (40.6%)	630 (40.3%)	793 (40.9%)	
One full-time caregiver	791 (22.6%)	350 (22.4%)	441 (22.8%)	
Intermediate one full-time caregiver and two or more full-time caregivers	115 (3.3%)	39 (2.5%)	76 (3.9%)	
Two or more full-time caregivers	35 (1.0%)	14 (0.9%)	21 (1.1%)	
Other	17 (0.5%)	9 (0.6%)	8 (0.4%)	
Unknown	115 (3.3%)	40 (2.6%)	75 (3.9%)	
Affected side				
Right	1,157 (33.0%)	495 (31.7%)	662 (34.2%)	<0.001
Left	1,176 (33.6%)	544 (34.8%)	632 (32.6%)	
Both	104 (3.0%)	67 (4.3%)	37 (1.9%)	
No paralysis	975 (27.8%)	399 (25.5%)	576 (29.7%)	
Unknown	89 (2.5%)	58 (3.7%)	31 (1.6%)	
Aphasia on NIHSS				
None	2,608 (74.5%)	1,103 (70.6%)	1,505 (77.7%)	<0.001
Mild to moderate	320 (9.1%)	131 (8.4%)	189 (9.8%)	
Severe	217 (6.2%)	94 (6.0%)	123 (6.3%)	
Mute, global aphasia	298 (8.5%)	197 (12.6%)	101 (5.2%)	
Missing	58 (1.7%)	38 (2.4%)	20 (1.0%)	
Gaze on NIHSS				
Normal	2,815 (80.4%)	1,139 (72.9%)	1,676 (86.5%)	<0.001
Partial gaze palsy	382 (10.9%)	212 (13.6%)	170 (8.8%)	
Forced deviation	234 (6.7%)	166 (10.6%)	68 (3.5%)	
Missing	70 (2.0%)	46 (2.9%)	24 (1.2%)	
Neglect on NIHSS				
Normal	2,589 (74.0%)	1,052 (67.3%)	1,537 (79.3%)	<0.001
Mild to moderate	408 (11.7%)	196 (12.5%)	212 (10.9%)	
Severe	433 (12.4%)	269 (17.2%)	164 (8.5%)	
Missing	71 (2.0%)	46 (2.9%)	25 (1.3%)	
No speech and language therapy	774 (22.1%)	335 (21.4%)	439 (22.7%)	0.390

^a^GCS, glasgow coma scale; ^b^rtPA, recombinant tissue plasminogen activator.

**Table 2:** Characteristics of eligible patients before and after inverse probability of treatment weighting (IPTW).

	Before IPTW			After IPTW		
	Non-Active occupational therapy group	Active occupational therapy group	Standardized difference	Non-Active occupational therapy group	Active occupational therapy group	Standardized difference
Continuous variable, Mean (SD)						
Age	70.2 (13.2)	68.3 (13.1)	−0.524	69.0 (13.5)	69.0 (13.0)	−0.009
Time from onset to admission (days)	1.3 (0.7)	1.4 (0.9)	0.090	1.3 (0.8)	1.3 (0.8)	0.000
Time to start of rehabilitation after admission (days)	3.0 (6.9)	2.5 (6.9)	−0.185	2.8 (6.6)	2.8 (8.6)	0.026
mRankin scale score	3.8 (1.4)	3.4 (1.4)	−0.349	3.6 (1.4)	3.6 (1.4)	0.013
GCS score	13.1 (3.3)	14.0 (2.2)	0.552	13.6 (2.8)	13.6 (2.7)	−0.023
NIHSS score	8.7 (9.6)	5.4 (6.6)	−1.140	6.8 (8.4)	7.0 (8.3)	0.051
Total FIM score	55.1 (34.6)	65.7 (32.7)	1.827	61.8 (34.9)	61.0 (33.2)	−0.136
Category variable, Proportion, %						
Female	44.0	37.5	−0.109	40.4	40.8	0.008
Type of stroke						
Ischaemic stroke	57.5	64.7	0.121	61.6	61.3	−0.005
Haemorrhagic stroke	26.6	23.1	−0.067	24.3	24.6	0.006
Subarachnoid haemorrhage	10.4	7.1	−0.098	8.8	8.9	0.004
Others, Unknown	5.4	5.1	−0.012	5.3	5.2	−0.004
rtPA treatment (yes)	3.1	1.4	−0.099	2.1	2.0	−0.007
Surgery after haemorrhagic stroke (yes)	8.3	5.0	−0.111	6.5	6.7	0.007
Surgery after subarachnoid haemorrhage (yes)	4.3	2.8	−0.069	3.5	3.8	0.014
Anticoagulant therapy						
No	69.0	74.5	0.101	71.8	72.5	0.014
Anticoagulant therapy (yes)	10.4	8.6	−0.053	9.9	8.8	−0.031
Antiplatelet therapy (yes)	9.7	9.2	−0.012	9.2	9.6	0.010
Anticoagulant therapy + Antiplatelet therapy	2.8	2.1	−0.038	2.5	2.2	−0.015
Unknown	8.1	5.6	−0.083	6.6	6.9	0.009
Number of Caregivers						
Almost none	30.8	27	−0.068	29.6	28.0	−0.029
Intermediate Almost none and one full-time caregiver	40.3	40.9	0.010	39.6	41.2	0.026
One full-time caregiver	22.4	22.8	0.007	22.9	22.2	−0.012
Intermediate one full-time caregiver and two or more full-time caregivers	2.5	3.9	0.064	2.3	4.2	0.084
Two or more full-time caregivers	0.9	1.1	0.015	0.8	1.1	0.026
Other	0.6	0.4	−0.020	0.7	0.3	−0.051
Unknown	2.6	3.9	0.059	4.1	3.0	−0.051
Affected side						
Right	31.7	34.2	0.043	32.6	32.6	−0.001
Left	34.8	32.6	−0.038	33.5	33.5	0.000
Both	4.3	1.9	−0.120	3.2	2.9	−0.014
No paralysis	25.5	29.7	0.076	27.6	29.3	0.030
Unknown	3.7	1.6	−0.115	3.1	1.7	−0.074
Aphasia on NIHSS						
None	72.0	78.5	0.125	76.9	75.5	−0.025
Mild to moderate	8.8	9.9	0.029	8.8	9.6	0.023
Severe	6.3	6.4	0.003	5.3	6.9	0.052
Mute, global aphasia	12.9	5.3	−0.235	9.1	8.0	−0.030
Gaze on NIHSS						
Normal	75.2	87.5	0.273	82.2	81.7	−0.011
Partial gaze palsy	13.9	8.9	−0.134	10.9	11.1	0.005
Forced deviation	10.8	3.6	−0.253	6.8	7.2	0.011
Neglect on NIHSS						
Normal	69.4	80.3	0.214	76.2	75.5	−0.013
Mild to moderate	13.0	11.1	−0.048	11.2	12.1	0.022
Severe	17.6	8.6	−0.234	12.6	12.4	−0.004
No speech and language therapy	21.4	22.7	0.024	22.5	22.3	−0.002

GCS, glasgow coma scale; rtPA, recombinant tissue plasminogen activator; NIHSS, national institutes of health stroke scale; FIM, functional independence measure; mRankin, modified rankin; SD, standard deviation; IPTW, inverse probability of treatment weighting.

### Outcomes and subgroup analysis

Table 3 shows the primary and secondary outcomes, total rehabilitation time, mean daily rehabilitation time, and mean daily time for each type of therapy. There was a significant difference in the mean daily rehabilitation time and daily speech and language therapy time between the AOT and non-AOT groups. Additionally, the total FIM score on admission indicated an absolute standardized difference of >0.1 after IPTW. Therefore, these variables were adjusted for in the final outcomes and subgroup analyses because they were potential confounding factors. The results of the multiple regression analysis adjusted for these variables showed that the AOT group had a significantly greater improvement in FIM and NIHSS scores and a significantly shorter length of hospitalization than the non-OT group (*p* < 0.001 for all). Meanwhile, the rate of discharge between the two groups did not differ significantly (*p* = 0.201).

**Table 3:** Intensity of rehabilitation and outcomes before and after inverse probability of treatment weighting.

	Before IPTW				After IPTW			
	Non-Active occupational therapy group	Active occupational therapy group	95% CI	*p*- value	Non-Active occupational therapy group	Active occupational therapy group	95% CI	*p*- value
Mean (SE)								
Total rehabilitation time, min	2376.6 (59.7)	1862.8 (39.9)	−654.6, −373.1	<0.001	2130.9 (56.4)	2035.4 (42.8)	−239.6, 48.7	0.195
Daily rehabilitation time, min	75.2 (1.3)	78.9 (1.0)	0.5, 6.8	0.025	72.7 (1.3)	79.9 (1.0)	4.0, 10.6	<0.001
Daily physical therapy time, min	36.9 (0.6)	24.6 (0.3)	−13.6, −10.9	<0.001	35.7 (0.6)	25.3 (0.3)	−11.8, −9.4	<0.001
Daily occupational therapy time, min	23.7 (0.5)	35.8 (0.5)	10.7, 13.4	<0.001	22.8 (0.5)	36.1 (0.5)	11.8, 14.6	<0.001
Daily speech and language therapy time, min	14.7 (0.4)	18.5 (0.4)	2.7, 4.9	<0.001	14.1 (0.4)	18.6 (0.4)	3.3, 5.6	<0.001
NIHSS score gain	2.8 (0.2)	2.3 (0.1)	−0.9, −0.1	0.006	2.2 (0.1)	3.0 (0.1)	0.3, 1.1	<0.001*
FIM score gain	31.0 (0.7)	35.8 (0.6)	3.0, 6.6	<0.001	31.3 (0.7)	35.8 (0.7)	2.0, 5.7	<0.001*
Length of hospital stay, days	30.8 (0.6)	23 (0.4)	−9.1, −6.4	<0.001	27.9 (0.5)	25.2 (0.4)	−3.7, −1.3	<0.001*
Rate of discharge to home, %	47.6	62.1		<0.001	54.9	56.9		0.201*

* Multiple regression of weighted data adjusting for unbalanced factors (standardized difference >0.1 after inverse probability of treatment weighting; unbalanced variables that occurred after the intervention).

Table 4 shows the effects of AOT on the FIM scores according to the patients' baseline characteristics after IPTW. We observed significant interactions for increased FIM scores of consciousness, stroke severity (NIHSS score on admission), ADLs (FIM score on admission), and aphasia, gaze, and neglect on the NIHSS. In terms of stroke severity, the more severe the limitation in performing ADLs and the worse the NIHSS scores for aphasia, gaze, and neglect, the higher the increase in FIM scores in the AOT group (*p* = 0.001–0.040). Regarding consciousness, the increase in FIM scores in the AOT group was significantly lower in patients with lower levels of consciousness (*p* = 0.004; GCS score ≥16 vs. <9).

**Table 4:** Adjusted coefficients [95% confidence intervals (CI)] and interactions of the active occupational therapy group in terms of the gain in functional independence measures (FIM) score according to baseline patient characteristics after inverse probability of treatment weighting.

Subgroup	Variable	Coefficient	95% CI	*p*-value	*p*-value for interaction
Sex	Male	5.6	3.3, 7.8	<0.001	ref
	Female	1.4	−1.6, 4.4	0.361	0.055
Age	≤54	2.1	−2.6, 6.7	0.375	ref
	55–64	2.3	−1.6, 6.2	0.246	0.949
	65–74	3.5	0.3, 6.7	0.033	0.785
	75–84	3.9	0.7, 7.1	0.017	0.553
	≥85	5.1	0.1, 10.0	0.044	0.425
Consciousness	GCS score 3–8	11.2	3.6, 18.8	0.004	ref
	9–13	5.4	0.2, 10.6	0.041	0.224
	≥14	3.0	1.4, 4.7	<0.001	0.004
Stroke Severity	NIHSS score <9	2.3	0.7, 3.9	0.006	ref
	10–15	7.3	2.2, 12.4	0.005	0.020
	≥16	8.0	3.1, 12.8	0.001	0.004
ADL	FIM motor score <50	4.9	2.5, 7.3	<0.001	ref
	50–69	0.2	−2.3, 2.7	0.868	0.010
	70–79	0.5	−3.1, 4.2	0.767	0.099
	80–84	−1.7	−7.8, 4.5	0.579	0.274
	≥85	3.1	−1.7, 8.0	0.199	0.426
Aphasia	None	2.1	0.4, 3.9	0.018	ref
	Mild to moderate	8.3	1.6, 15.1	0.016	0.009
	Severe	7.4	−0.5, 15.3	0.065	0.016
	Mute, global aphasia	6.0	−0.6, 12.6	0.077	0.040
Gaze	Normal	2.5	0.7, 4.2	0.006	ref
	Partial gaze palsy	9.7	3.7, 15.7	0.002	0.001
	Forced deviation	11.5	4.0, 19.0	0.003	0.003
Neglect	Normal	2.0	0.3, 3.8	0.024	ref
	Mild to moderate	8.4	2.6, 14.1	0.004	0.002
	Severe	9.2	3.7, 14.7	0.001	0.001

GCS, glasgow coma scale.

Multiple regression of weighted data adjusting for unbalanced factors (standardized difference >0.1 after inverse probability of treatment weighting; unbalanced variables that occurred after the intervention).

## Discussion

This retrospective cohort study investigated how AOT affected patients with acute stroke while replacing missing values using multiple imputation and IPTW to adjust for confounding factors. Additionally, we conducted a subgroup analysis using the data after IPTW to identify patient characteristics associated with effective AOT. The results suggested that AOT was more effective than non-AOT in improving the limitations in performing ADLs and reducing the length of hospitalization of patients with acute stroke. The subgroup analysis results also indicated that AOT was more effective in patients with severe limitations in performing ADLs and cognitive impairment, such as neglect, on admission.

A previous study reported that the total duration of OT significantly influenced the FIM scores of patients with stroke in a rehabilitation unit (36). Furthermore, OT resulted, for patients with stroke and those with other illnesses, in reduced readmission rates (37, 38). Occupational therapists primarily engage patients in activities targeted at improving their ability to perform ADLs using an approach aimed at reducing impairment and improving function (18). The results of this study confirmed those of previous studies and were also novel in that they revealed the effects of intensive OT during the acute phase of stroke; however, OT is administered less commonly than physical therapy during this phase, with only 61% of patients receiving both physical therapy and OT (39). We believe that intervention with OT in the acute phase of stroke to improve limitations in performing ADLs may increase the FIM scores and reduce the length of hospitalization in patients, as revealed in this study. AOT is widely available and, according to our results, should be recommended for patients with acute stroke.

There are several limitations to this study. First, the duration of daily rehabilitation and speech and language therapies differed between the two groups; however, the difference in the daily rehabilitation times between groups was only approximately 7 min, which is unlikely to have a substantial effect. Furthermore, the final analysis involved adjustment for this covariate. Second, the difference in outcome scores between the two groups was small (4.5 points for the FIM gain score and 0.8 points for the NIHSS score) and should be interpreted with caution; however, compared with those of previous studies, the observed differences in these scores would be considered sufficient (17, 36). Furthermore, in the subgroup analysis, there was a difference of nearly 10 points between the two groups in FIM gain score, suggesting that AOT should be provided according to patient characteristics. Finally, although we adjusted for 18 potential confounders affecting the main outcome, including those related to the patients' age, level of consciousness, and stroke severity on admission, unmeasured confounders could have remained in the multivariate logistic regression calculation of the propensity score. In the future, randomized controlled trials that strictly adjust for confounding factors should be conducted to definitively determine the efficacy of AOT in treating patients with acute stroke.

The results of this study indicate that AOT improved the limitations in performing ADLs and physical function as well as reduced the length of hospitalization of patients with acute stroke. Additionally, the results of the subgroup analysis suggested that AOT was more effective for patients with severe limitations in performing ADLs and cognitive impairment, such as neglect, on admission. OT should be widely recommended for patients with acute stroke.

## Data Availability

The datasets presented in this article are not readily available because of the confidential data provided by the Japan Association of Rehabilitation Database. Therefore, this data could not be shared. Requests to access the datasets should be directed to the corresponding author.
