# Supplementary figures and images for: Effectiveness of active occupational therapy in patients with acute stroke: A propensity score-weighted retrospective study
Source: Front Rehabil Sci. 2023 Jan 5;3:1045231. doi: 10.3389/fresc.2022.1045231 (PMC9849931; doi:10.3389/fresc.2022.1045231)

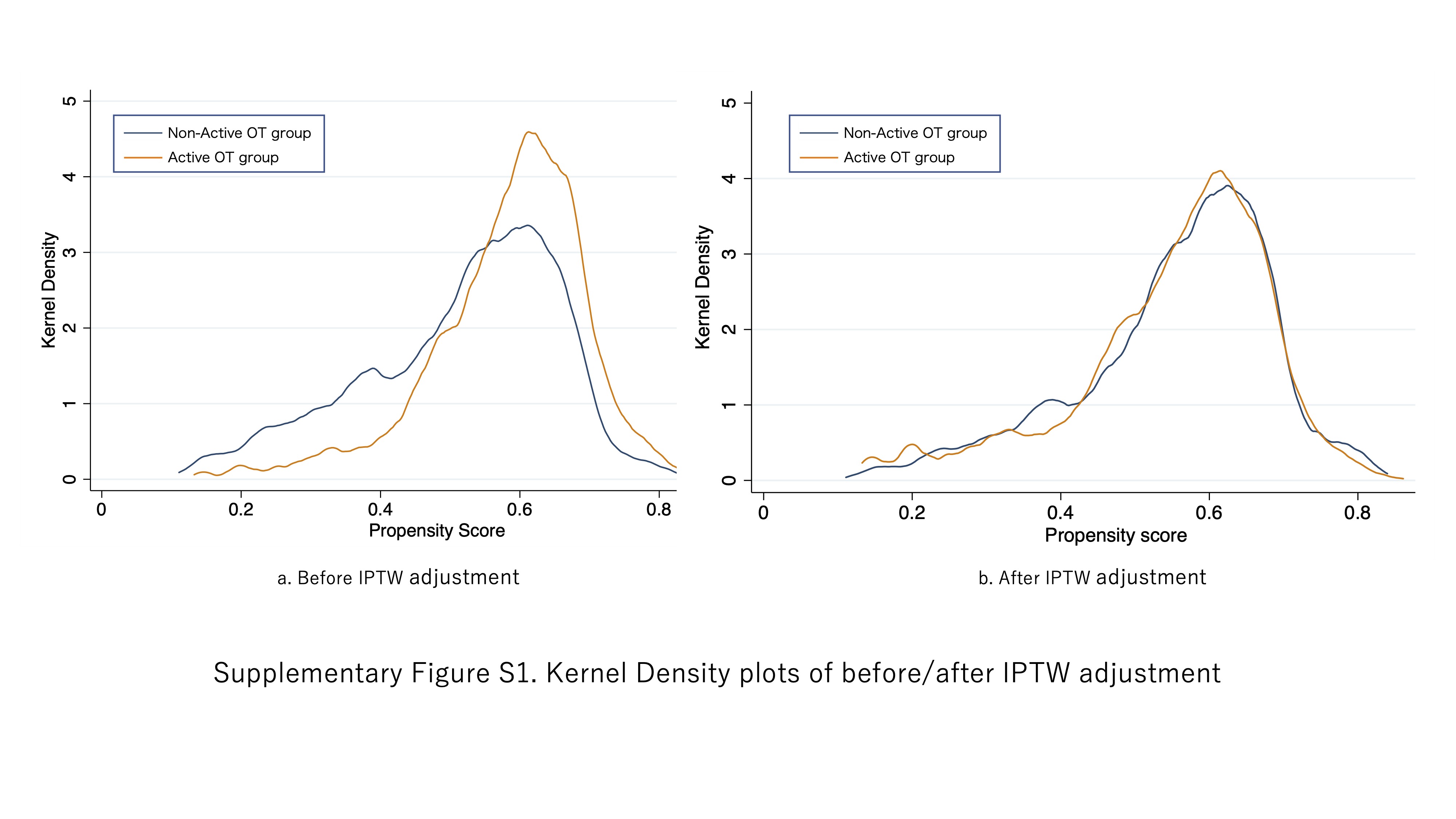

Supplement: Supplementary file 1 [file Image1.jpg]

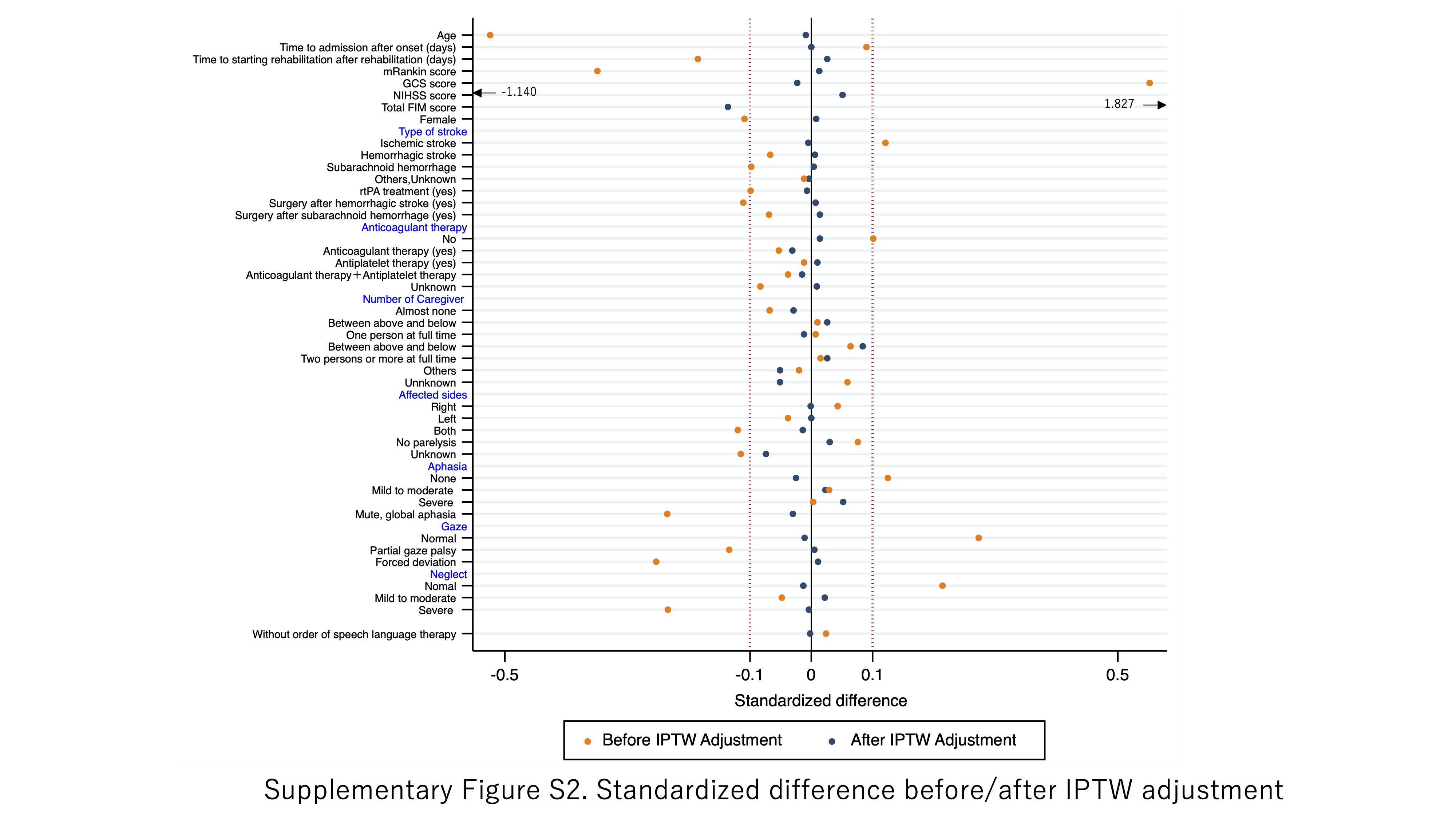

Supplement: Supplementary file 2 [file Image2.jpg]
